# Identifying Genetic Risk Factors for Diabetic Macular Edema and the Response to Treatment

**DOI:** 10.1155/2020/5016916

**Published:** 2020-11-12

**Authors:** Rajya L. Gurung, Liesel M. FitzGerald, Bennet J. McComish, Nitin Verma, Kathryn P. Burdon

**Affiliations:** ^1^Menzies Institute for Medical Research, University of Tasmania, Hobart, TAS, Australia; ^2^School of Medicine, University of Tasmania, Hobart, TAS, Australia

## Abstract

Diabetic retinopathy (DR) is the most common microvascular complication of diabetes mellitus (DM). DR is complex and the term encompasses several clinical subtypes of diabetic eye disease, including diabetic macular edema (DME), the most frequent cause of central vision loss in DR patients. Both genetic and environmental factors contribute to the pathophysiology of DR and its subtypes. While numerous studies have identified several susceptibility genes for DR, few have investigated the impact of genetics on DME susceptibility. This review will focus on the current literature surrounding genetic risk factors associated with DME. We will also highlight the small number of studies investigating the genetics of response to antivascular endothelial growth factor (anti-VEGF) injection, which is used to treat DME.

## 1. Introduction

Diabetic retinopathy (DR), the most common microvascular complication of diabetes mellitus (DM), is a leading cause of vision loss in the working-age population [1]. It is a heterogeneous condition with multiple subtypes. DR can present as mild nonproliferative retinal changes anywhere in the retina having little or no effect on vision, to severe nonproliferative retinopathy characterized by severe retinal hemorrhages and vascular changes. A portion of patients will progress to proliferative retinopathy, characterized by aberrant neovascularization. This has a profound effect on vision, leading to permanent vision loss or blindness [2]. Diabetic macular edema (DME) is another retinal complication of diabetes and is often included under the umbrella of DR. It can occur at any stage of the progression from nonproliferative to proliferative disease, with or without other features of DR [3] and in conjunction with type 1 (T1) or type 2 (T2) DM. It is the most frequent cause of central vision impairment in patients with diabetes [4] with a reported global prevalence of 4.6% amongst diabetics between 2015 and 2019 [5]. DME presents as a collection of fluid in the central part of the retina, mainly in the inner and outer plexiform layers. It can be associated with hard exudates, which present clinically as yellow-white plaque deposits in the macular region. The gold standard and most widely used classification of DME is clinically significant macular edema (CSME), defined by the Early Treatment for Diabetic Retinopathy Study (ETDRS) as (1) retinal thickening at or within 500 microns of the macular center; (2) hard exudates at or within 500 microns of the macular center, associated with adjacent retinal thickening; or (3) one or more disc diameter of retinal thickening, any part of which lies within one disc diameter of the macular center [6]. DME is often included in the broader classification of diabetic maculopathy, which also includes diabetic macular ischemia [7]. It should also be noted that many studies do not necessarily consider DME separately from the larger collective of DR phenotypes.

From studies to date, primarily under the umbrella phenotype of DR, it appears that conventional risk factors like diabetes duration, poor glycemic control, hyperlipidemia, microalbuminuria, and high diastolic blood pressure explain only a small portion of the risk for development and progression of diabetic microvascular complications, including DME [8, 9]. Moreover, a significant proportion of participants remain free of diabetic complications or progression even after a long disease duration [10]. Thus, other factors, including genetics, likely contribute to DR and DME risk.

The genetics of DR has been studied extensively in the last decade; however, most of these studies failed to distinguish DME as a separate phenotype of DR. The majority of studies that have made this distinction consisted of small sample sizes, limiting statistical power. Here, we review the literature related to the genetics of DME. The limitations of these studies are discussed and our current understanding of the genetic architecture of DME is summarized. In addition, we discuss the studies that have evaluated genetic factors involved in a patient's response to antivascular endothelial growth factor (anti-VEGF) injection. This group of drugs is now a frontline treatment for DME, but patient outcomes remain mixed, and understanding this variability is critical if we wish to improve outcomes. Articles published in English before January 2020 were identified through searches of PubMed, Embase, and Web of Science. Also, we manually searched the reference lists of included papers to identify other potentially eligible studies. Case reports, editorials, abstracts, reviews, and unpublished reports were excluded. A total of 61 genetic studies had DME/diabetic maculopathy mentioned in their study, of which 48 specifically state that their cohort included DME patients. Of these 48, only 24 studies conducted a separate analysis for DME.

## 2. Candidate Genes

The candidate gene approach focuses on establishing a genetic association between predefined genes and disease status or phenotypes [11]. Genes are selected based on prior knowledge of the molecular pathways underlying the pathophysiology of a disease and the known or presumed function of the gene in those pathways. To date, less than a dozen candidate genes have been found to be associated with DME, and findings for most have been variable (Table 1).

### 2.1. Apolipoprotein E (*APOE*)

In DME, macular exudates contribute to significant visual loss when present in the foveal center and are frequently associated with a high level of serum lipids [12, 13]. Apolipoprotein E (APOE) is mainly known for lipid transportation and metabolism. It is highly expressed in the retina and has been explored as a possible DME susceptibility gene. The gene is polymorphic with three major alleles; epsilon 2 (*ε*2), epsilon 3 (*ε*3), and epsilon 4 (*ε*4) [14]. Santos et al. [15] conducted a study on 36 T2 DME patients (compared to 22 healthy individuals) to determine the relationship between *APOE* polymorphisms and the severity of macular edema. DME severity was graded based on the number and extent of macular hard exudates using standardized retinal photographs based on the Early Treatment Diabetic Retinopathy Study (ETDRS) guidelines [16]. In their study, the frequency of macular hard exudates was higher in *ε*4 carriers (*p* < 0.05). However, there was poor correlation between degree of visual impairment and presence of the *ε*4 allele (*p* = 0.057). Estimation of lipid levels found significantly higher total lipids in the *ε*4 carrier group (*p* < 0.05). Given the small sample size with borderline significant results, extreme caution should be taken when interpreting this study and much larger studies are required to draw conclusions about the role of *APOE* variants in DME.

### 2.2. Nitric Oxide Synthase (*NOS*)

Damage to vascular endothelial cells can lead to exudation of fluid into the retinal space, a hallmark feature of DME. Damage can be caused through a range of mechanisms, including oxidative damage from free radicals. One such molecule is nitric oxide, produced by the conversion of L-arginine to L-citrulline by nitric oxide synthase (NOS) [17]. There are three recognized isoforms of NOS: the constitutively expressed, neuronal NOS (nNOS/NOS-1), endothelial NOS (eNOS/NOS-3), and the inducible NOS (iNOS/NOS-2), upregulated in response to stimuli [17]. The isoform *eNOS* has been posited as a candidate gene for DME due to its role in endothelial cells of the vasculature. The most commonly studied polymorphisms in eNOS are: -786T>C (rs2070744) in the promoter region; 894G>T (rs1799983) substitution in exon 7; and a 27-bp variable number tandem repeat in intron 4 with “a” and “b” alleles that differ in their number of repeats (27-bp*VNTR* (a/b)). Only two studies to date have analyzed *eNOS* gene polymorphisms specifically for association with DME. Awata et al. [18] studied *eNOS* gene polymorphisms in a Japanese cohort of T2DM patients compared with healthy controls. Subgroup analysis of DME patients (DME = 48, DRwithoutDME = 69) revealed that the -786T>C polymorphism and 27-bp *VNTR* were significantly associated with the risk of developing DME. Specifically, the -786C allele (*p* = 0.029) and the 27-bp *VNTR* “a” allele (*p* = 0.006) appeared to increase the risk of DME, with significantly different genotype frequencies between the cohort with and without DME. The results were consistent when clinical covariates were also included in the analysis model (*p* = 0.001, OR = 3.57, 95%CI = 1.65–7.69). The 894G>T polymorphism was not associated with DME risk in either the allelic or genotypic model. In a similar study, Uthra et al. [19] tested the association between the 27-bp *VNTR* and DR in a South Indian T2DM cohort but did not identify any significant association. In a subgroup analysis, the frequency of genotypes and alleles of the 27-bp *VNTR* was compared between DR with, (*n* = 100) and without DME (*n* = 87), but no significant association with DME was observed (*p* > 0.05) [19]. Thus, there are conflicting reports for this gene and further larger studies are required for a better understanding of the role of the e*NOS* gene in the pathogenesis of DME.

### 2.3. Manganese Superoxide Dismutase (*SOD2*)

Another gene involved in oxidative stress is *SOD2*, encoding manganese superoxide dismutase (MnSOD) [20]. This enzyme protects against the damaging effects of superoxide radicals, which are postulated to trigger several biochemical pathways underlying the pathophysiology of DR and DME [21]. The Ala16Val (rs4880) polymorphism in *SOD2* results in a 30-40% lower enzymatic activity of MnSOD and hence a greater cellular susceptibility to oxidative stress [22]. This polymorphism has been studied in association with DR risk in different cohorts across many ethnicities and countries [23, 24]. A study by Lee et al. [25] in a Korean T2DM cohort is the only one to explore an association between the Ala16Val polymorphism and DME. The DME subgroup (*n* = 37) was found to have a significantly lower Ala allele frequency (*p* < 0.05) when compared to the non-DME group (DR without DME = 93). In multivariate logistic regression, the Ala allele of *SOD2* was associated with DME (*p* = 0.03, OR = 1.59, 95%CI = 1.02–2.02). Further, disparate Ala allele frequencies were observed in the three DME subtypes; focal = 0.188 (*n* = 8), diffuse = 0.109 (*n* = 23), and ischaemic = 0.0 (*n* = 6); however, this could not be statistically evaluated due to the small sample size. Overall, due to the limited numbers of DME patients in this study, the results should be interpreted with caution and additional better-powered studies need to be undertaken to determine whether *SOD2* has a role in DME risk.

### 2.4. Erythropoietin (*EPO*)

Human erythropoietin (EPO) is a potent angiogenic factor secreted in response to hypoxia by a mechanism dependent upon the hypoxia-inducible factor (HIF). EPO regulates the production of red blood cells via its receptor, erythropoietin receptor (EPOR), which is expressed in retinal tissue [26]. There are several studies demonstrating the protective role of EPO in maintaining the integrity of the blood-retinal barrier, the structure primarily responsible for the pathogenesis of DME [27–29], thus making *EPO* an important candidate gene. Abhary et al. [30] genotyped *EPO* gene polymorphisms in both T1 and T2 diabetic patients exhibiting different severity levels of DR. In this study, the GG (rs1617640), CC (rs507392), and CC (rs551238) genotypes were found to be associated with increased risk of DR in the combined DM group (*p* = 0.008), and the T2DM group alone (*p* = 0.006). This study also analyzed *EPO* polymorphisms in the DME cohort separately. All three *EPO* SNPs were associated with DME in the combined DM group (DME (*n* = 90) vs. DM without DR (*n* = 233), *p* = 0.04) as well as in the T2DM only group (DME (*n* = 66) vs. DM without DR (*n* = 166), *p* = 0.018). Additionally, the GCC haplotype of all three SNPs was significantly associated with DME both in the combined DM group (*p* = 0.04) and T2DM alone (*p* = 0.031). Analogous reports of positive *EPO* genotype associations with DR have been presented by several other studies [31, 32]; however, only Abhary et al. [30] study analyzed DME separately. Taken together, there is evidence that *EPO* has a role in DR, but a larger cohort of DME patients should be assessed to replicate the DME-specific findings.

### 2.5. Vascular Endothelial Growth Factor-A (*VEGFA*)

The vascular endothelial growth factor (VEGF) family is a group of five structurally related glycoproteins [33]: VEGF-A, VEGF-B, VEGF-C, VEGF-D, and placental growth factor (PGF), each encoded by a separate gene corresponding to their respective names. VEGF-A, commonly referred to as VEGF, is mainly responsible for vasculogenesis (formation of new blood vessels during embryogenesis) and angiogenesis (formation of new blood vessels from preexisting blood vessels) [34]. Both serum and vitreous VEGF protein levels are significantly elevated in diabetic compared to nondiabetic individuals, and anti-VEGF agents are the latest standard of care for the management of DME [35]. The *VEGFA* gene is highly polymorphic and extensively studied in relation to DR; however, its role in DME risk is relatively unexplored. Of the seven studies on *VEGFA* polymorphism related to DR [36–40], only three have analyzed DME patients separately.

Awata et al. [37] studied three polymorphisms in the promoter and upstream region of the *VEGFA* gene (-2,578C>A [rs699947], -1,154G>A [rs1570360], and -634C>G [rs2010963]) in a cohort of T2 diabetic patients. In the subgroup analysis (DME = 63, DRwithoutDME = 112), the frequencies of both the -634C>G CC genotype (*p* = 0.023) and C allele (*p* = 0.023) were significantly increased in DME patients. The CC genotype remained significantly associated with DME risk after adjusting for clinical covariates (*p* = 0.047, OR = 1.81, 95%CI = 1.01–3.26). Furthermore, macular thickness measured by optical coherence tomography was also found to be correlated with the same allele (*p* = 0.006), independent of the duration of diabetes. There were no significant differences in the genotype and allele frequencies of -2,578C>A or -1,154G>A in the overall cohort analysis or any subgroup analyses.

More recently, Shazly et al. [40] undertook a similar analysis of the *VEGFA* -634C>G polymorphism in a cohort of T2 diabetic patients. In the subgroup analysis for DME (DME = 64, DRwithoutDME = 148), they observed a significant association between DME risk and the CC genotype (*p* = 0.019) and C allele (*p* = 0.022), corresponding to the genotypic and allelic model, respectively. Multivariate logistic regression, taking into account both genetic and clinical covariates, also identified the CC genotype of the -634C>G polymorphism as a significant risk variant for DME (*p* = 0.003). However, a subgroup analysis dividing DME patients according to the proliferative state of DR (nonproliferativeDR = 20, proliferativeDR = 44) failed to show any significant association in either of the models assessed in this small cohort. Interestingly, the CC genotype was associated with a significantly higher serum concentration of VEGF in DME patients (*p* = 0.016).

Using a slightly different approach, Abhary et al. [36] investigated the association between 15 *VEGFA* tag SNPs with DME in a cohort of diabetic patients (T1+T2). The minor allele of rs699946 (A; *p* = 0.039, OR = 5.7, 95%CI = 1.1–29.3) and rs833068 (G; *p* = 0.017, OR = 5.1, 95%CI = 1.3–19.5) were significantly associated with DME risk in T1DM. In T2DM, the G allele of rs10434 was associated with DME (*p* = 0.027, OR = 2.9, 95%CI = 1.1–7.6). Combined analysis for both types of DM (DME = 93, DMwithoutDR = 281) found a significant association of DME with the G allele of rs10434 (*p* = 0.003). This result remained significant after correcting for multiple testing (*p* = 0.03). Thus far, studies on *VEGF* polymorphisms in association with DME show encouraging results. Although the cohorts of DME patients are relatively small, associations of *VEGFA* SNPs with DME are consistently observed across studies. Given the known role of VEGFA in DME pathogenesis and the success of treatments that target this protein, these results are not unexpected. The relative contribution of this gene to the overall risk profile of DME remains to be determined and larger studies to better investigate the true effect size would be warranted.

### 2.6. Vascular Endothelial Growth Factor-C (*VEGFC*)

VEGF-C is a dimeric glycoprotein of the VEGF family encoded in humans by the *VEGFC* gene. Along with VEGF-D, it acts as a major lymphangiogenic factor, leading to the formation of new lymph vessels [41] by binding to VEGF receptor 3 (VEGFR-3). Though the eye was historically considered to lack a lymphatic system, recent studies suggest lymphangiogenesis may have a role in macular edema [42]. Furthermore, VEGF-C is believed to promote retinal neovascularization independent of its widely reported counterpart VEGF-A [43, 44]. Interestingly, VEGF-C and its receptor VEGFR-3 are markedly increased in the retinal vessels of DR patients [45]. To date, only one study has investigated genetic polymorphism in the *VEGFC* gene and its association with DME risk [46]. Kaidonis et al. [46] investigated the association of 13 *VEGFC* tag SNPs with DR risk in Caucasian diabetics (T1+T2). In the overall analysis including “any DR” across both types of DM, three *VEGFC* SNPs (rs17697515, rs17697419, and rs2333526) were significantly associated with DR risk even after adjustment for clinical covariates and multiple testing. Further analysis stratified by diabetes type resulted in similar trends of association with the above-mentioned SNPs. In subset analyses of DME, the T allele of rs17697515 was negatively associated with DME risk in T2DM patients (DME = 361, DRwithoutDME = 711, *p* = 0.004, OR = 0.53, 95%CI = 0.35–0.82); however, no associations were detected in T1DM patients (DME = 64, DRwithoutDME = 241), and only a nominal association between rs17697515 and DME risk (DME = 425, DRwithoutDME = 952, *p* = 0.009) was observed in combined DM patients after correcting for multiple testing. Thus, evidence to date suggests that *VEGF-C* might play a role in DME pathogenesis along with *VEGFA*, but the exact mechanism(s) of action is not yet fully elucidated. Further, despite a large cohort of DME, there was only a nominal association; thus, the results of this study need to be replicated.

### 2.7. Pigment Epithelium-Derived Factor (*PEDF*)

Another candidate gene involved in angiogenesis pathways is pigment epithelium-derived factor (*PEDF*). PEDF, also known as serpin F1 (SERPINF1), is widely expressed in many organs and tissues, including the retinal pigment epithelial layer of the retina [47]. It is a member of the serine proteinase inhibitor (SERPIN) family, widely known as the most potent natural antiangiogenic factor [48]. In the retina, *PEDF* is known to inhibit and downregulate proangiogenic factors [49], and an imbalance between VEGF and PEDF in the vitreous has been implicated as one mechanism responsible for the development and progression of DME. To date, only one study, by Iizuka et al. [50], has investigated the association of *PEDF* gene polymorphisms with DME risk. In this study, DR cases were compared with diabetics without DR. The C allele of rs12150053 and A allele of rs12948385 were associated with DR risk in dominant and codominant models but were also observed to be associated with DME risk in a subgroup analysis (DME = 66, DMwithoutDR = 229, *p* < 0.05). It should be noted that there is strong linkage disequilibrium between these two polymorphisms. Subsequently, two studies by Uthra et al. [51] and Yamagishi et al. [52] evaluated *PEDF* gene polymorphisms with DR risk in Asian cohorts. Uthra et al. [51] observed a moderately protective association between a polymorphism in exon 4 (Thr130Thr) and DR risk, whilst Yamagishi et al. [52] failed to observe any association between a polymorphism in exon 3 (Met72Thr) and DR risk. Neither study performed subset analyses for associations with DME risk. As only one study to date has observed a significant association between DME and *PEDF* polymorphisms, the findings require replication in additional, larger datasets.

### 2.8. Aldose Reductase (*ALR2*)

Aldose reductase (ALR2), also known as aldo-keto reductase family 1 (AKR1B1/ALDR1), catalyzes the first rate-limiting step during glucose metabolism in the polyol pathway. Hyperglycaemia in DM leads to altered activity of *ALR2* and accumulation of sorbitol, which is responsible for various complications related to the disease [53]. Various polymorphic variants of the *ALR2* gene have been linked to the development of microvascular complications related to DM. Of note, the (CA)n dinucleotide repeat has been studied extensively for association with DR susceptibility across many ethnicities [54, 55]. The CA dinucleotide repeat has three common alleles consisting of 24 repeats (labeled the Z allele), 23 repeats (the Z-2 allele), and 25 repeats (the Z+2 allele). These allelic polymorphisms have been hypothesized to alter *ALR2* mRNA levels and hence enzyme activity, thus contributing to diabetic microvascular complications [56]. However, to date, conflicting evidence has been presented; some studies have reported an association between the Z-2 allele and DR risk [57, 58], whereas others have reported no association [54, 55]. In a meta-analysis by Mi et al. [57], comprising 17 studies, the Z-2 allele was reported as a risk polymorphism for DR in both Asian and Caucasian T1 and T2DM cohorts. Notably, only one study by Kumaramanickavel et al. [59] has explored the association between the *ALR2* dinucleotide repeat and DME risk. They evaluated a South Indian T2DM population and reported that the Z-2 allele showed a significant association with overall DR risk (*p* = 0.029). DR patients were then subclassified into “proliferative DR+maculopathy” (*n* = 20), “nonproliferative DR+maculopathy” (*n* = 35), “proliferative DR” (*n* = 35), and “nonproliferative DR” (*n* = 15). There was significant difference in the Z-2 allele frequency in the “proliferative DR+maculopathy” when compared with the proliferative DR (*p* = 0.004) and nonproliferative DR (*p* = 0.002) groups, but not when compared with the nonproliferative DR+maculopathy group. This study has attempted a detailed stratified analysis by various subtypes of DR involving DME but was unable to identify a robust association. Although meta-analysis suggests this variant is associated with DR risk, its role in DME specifically is yet to be elucidated.

### 2.9. MicroRNA Genes (*miRNA*)

MicroRNAs (miRNAs or miRs) are a class of short, noncoding, single-stranded RNA molecules responsible for regulating a plethora of biological processes [60]. Several miRNAs have been reported to be expressed in the retina, and their dysregulation has been linked to various retinal disorders [61]. Moreover, miRNAs have been shown to play a significant role in angiogenesis and oxidative stress [62] and have thus been proposed as biomarkers of DR and disease progression [63]. To date, very few studies have explored whether genetic variation in microRNAs is associated with DME risk. Kaidonis et al. [64] were the first group to report an association between microRNA-146a (miR-146a) and DME. The miR-146a SNP, rs2910164, was tested for association with microvascular complications in both T1 and T2DM patients. A subgroup analysis found an association between the C allele of rs2910164 and DME risk (DME = 856, no/minimalDR = 895) in the T2DM cohort (*p* = 0.025, OR = 1.25, 95%CI = 1.03–1.53). However, there was no association with T1 DME (DME = 170, no/minimalDR = 258) or proliferative DR.

A more recent study investigating the relationship between microRNA genes and DR risk was conducted by Liu et al. [65]. Imputed SNP array data was extracted from a previous T1DM GWAS [66] and tested for association with different DR phenotypes, including DME. No SNPs reached genome-wide significance for any of the subtypes of DR; nevertheless, the top SNPs from the proliferative DR and sight-threatening DR analyses were genotyped in a second cohort and the data from both samples combined. SNP rs10061133 in *MIR449b* was found to be protective against sight-threatening DR (*p* = 3.68 × 10^−4^, OR = 0.32, 95%CI = 0.17–0.60) and proliferative DR (*p* = 8.12 × 10^−4^, OR = 0.30, 95%CI = 0.15–0.61). The sight-threatening DR phenotype included DME patients as well as proliferative DR, with significant overlap of phenotype between patients, but the number of patients with DME was small compared to the number with proliferative DR (totalsight − threatening = 223, DME = 89, proliferativeDR = 181, DMwithoutDR = 228). Given the much larger number of patients with proliferative DR, the association signal was assumed to be driven by these patients.

In another study, McAuley et al. [67] found a significant association between a polymorphism in miRNA-126 and sight-threatening DR. The A allele of rs4636297, known to be the nonfunctional allele for posttranslational regulation of miR-126, was associated with severe sight-threatening DR (*p* = 0.006, OR = 2.02, 95%CI = 1.22–3.35). However, DME was not included in their definition of sight-threatening DR. Larger studies specifically evaluating the role of these miRNA variants in DME risk are required to confirm and replicate.

### 2.10. Other Candidate Genes

Several other genes have been studied in relation to DME risk but failed to show an association. Abhary et al. [68] investigated *carbonic anhydrase* (*CA*) sequence variation as a risk factor for DME (DME = 93/DMwithoutDR = 235) but found no associations in their cohort. Dong et al. [69] investigated a *monocyte chemoattractant protein-1* (*MCP1*) polymorphism in an Asian DM cohort, and while they observed a significant association with overall DR risk and proliferative DR, they found no association with severity of DME (mildDME = 207, moderateDME = 173, severeDME = 66). Another study by Dong et al. [70] using the same cohort of patients as above [69], analyzed an association between DR susceptibility and polymorphisms in the CXC chemokine family genes, *interleukin 8* (*IL8* or *CXCL8*; -251T>A) and *interferon gamma-induced protein 10* (*IP10* or *CXCL10*; -1596C>T) but failed to observe a significant association in a subgroup analysis of DME patients. Finally, Upadhyay et al. [71] studied the association between the *sarcolemma-associated protein* (*SLMAP*) gene polymorphism, rs17058639, and DR risk. Interestingly, in the subgroup analysis of DR, they did report a significant association of rs17058639 with DME (*p* = 0.0425), but the sample size was very small (DME = 49, DMwithoutDR = 160) and this result may be a false positive.

## 3. Genome-Wide Association Studies (GWAS)

Genome-wide association studies (GWAS) are aimed at identifying differences in the frequency of common genetic variants across the entire genome between groups of individuals. This technique has revolutionized the field of complex disease genetics [72]. Unlike the candidate gene approach, which depends on an *a priori* hypothesis, GWAS is considered a powerful hypothesis-free tool to identify genotype-phenotype associations and discover associations with variants in genes that have not been previously considered [72]. To account for the heavy burden of multiple hypothesis testing in a GWAS, the threshold for statistical significance is usually set at 5 × 10^−8^ when common variants with a population frequency > 5% are analyzed [73]. Small studies will only have the power to reach this stringent threshold when the effect size is very large. However, for many complex diseases, including DME, the expected effect size for most variants is quite low, requiring very large sample sizes to detect. To date, a total of 13 GWAS related to DR risk have been published, of which only two specifically focussed on the DME phenotype [74, 75]. The largest GWAS where DME risk was considered was reported by Meng et al. [75]. This study was done in a well-defined Scottish cohort of T2DM involving 469 DME cases (defined as diabetic maculopathy with decreased visual acuity) and 1,374 controls (DM without DR or maculopathy). A SNP in the *TTC39C* gene, rs9966620, reached genome-wide significance with a *p* value of 4.13 × 10^−8^ (OR = 1.95, 95%CI = 1.53–2.47). Two nearby SNPs, in linkage disequilibrium with rs9966620, also approached significance (rs7243626, *p* = 5.64 × 10^−8^, and rs7240470, *p* = 8.05 × 10^−7^). However, a GWAS considering a broader DME phenotype (maculopathy irrespective of vision loss, *n* = 1,240) failed to identify any SNPs reaching genome-wide significance. Whilst the *TTC39C* gene product is expressed in the eyes, the function of the protein is yet to be elucidated.

Another GWAS related to DR by Graham et al. [74] performed analyses for DME (DME = 270, DMwithoutDR = 435) and proliferative DR. The authors found no genome-wide significant associations with DME risk. Their two highest hits were rs1990145 (*p* = 4.10 × 10^−6^, OR = 2.02, 95%CI = 1.50–2.72) and rs4771506 (*p* = 6.94 × 10^−6^, OR = 1.97, 95%CI = 1.46–2.64). The SNP rs1990145 is located in an intron of the *MRPL19* gene on chromosome 2 and rs4771506 is on chromosome 13 near the *LINC00343* gene. Further, this study also evaluated the top SNPs reported in a previous DR GWAS study (T1DM) by Grassi et al. [76]. Two SNPs reported in that study to be associated with DR, rs12267418 near *MALRD1* and rs16999051 within *PCSK2* on chromosome 20, were found to be nominally associated with DME (*p* = 0.008 and *p* = 0.007, respectively). Whilst the above studies provide some evidence for possible novel candidate DME risk genes, given the size of most of the cohorts, these findings need to be replicated in larger studies.

## 4. Genetic Predictors of Treatment Response

Based on the Early Treatment Diabetic Retinopathy Study (ETDRS), macular laser photocoagulation was the gold standard treatment for managing DME for many years [6]. However, intravitreal injections of VEGF inhibitors have now revolutionized the management of DME. Today, anti-VEGF intravitreal injection with or without adjunct focal laser is the standard of care for treating center-involving DME in most countries [77]. Three commonly used intraocular anti-VEGF agents are aflibercept (Eylea, Regeneron Pharmaceuticals), bevacizumab (Avastin, Genentech), and ranibizumab (Lucentis, Genetech) [77]. Despite the widespread use of anti-VEGF agents, there is wide variability in patient outcomes. This variability was initially apparent in clinical trials, where a significant proportion of patients failed to achieve a functional or anatomical response [78] but is even more striking in the real-world clinical setting [79]. While some of these variations in treatment response can be explained by clinical and environmental factors, it has been postulated that inherited genetic variation may also play a role. Many post hoc analyses from clinical trials [80, 81] and real-world clinical studies [79, 82] have attempted to identify ocular and systemic predictors of response to anti-VEGF treatment. The relationship between genetic variation and response to anti-VEGF has been studied quite extensively in other disorders that use these drugs, including age-related macular degeneration (AMD) [83] and cancer [84], but only four studies (Table 2) have specifically investigated genetic differences between responders and nonresponders to anti-VEGF injections in DME patients [40, 85–87].

Shazly et al. [40] is the only group to report a significant correlation between a patient's genetic profile and response to anti-VEGF (bevacizumab) therapy in DME. The response arm of the study (mentioned above in the Candidate Genes section) involved 64 DME patients. The distribution and allele frequency of the *VEGFA* -634C>G polymorphism (rs2010963) was compared between poor (*n* = 24) and good (*n* = 40) responders. Response was defined based on the change in best-corrected visual acuity and central macular thickness (Table 2) and patients were followed up every month for 9-12 months. The -634C>G polymorphism was selected due to its strong association with DME and DR from previous studies [37, 88]. The study identified a significantly higher CC genotype frequency amongst good responders compared to the poor responder group (*p* < 0.001), even after adjusting for clinical and demographic covariates. Likewise, the frequency of the -634C allele was significantly higher in the good responders compared to the poor responders (*p* < 0.001).

A study by Tetikoglu et al. [85] also investigated *VEGFA* gene polymorphisms (rs2010963, rs2146323, rs10434, rs833069, and rs6921438) and their association with response to intravitreal ranibizumab treatment. The response criteria in this study were less stringent (two lines improvement in visual acuity compared to three lines in Shazly et al.) and the 95 DME patients (goodresponders = 53, poorresponders = 42) were followed up for only 5 months. Despite a significant difference in visual outcome clinically (*p* = 0.001), there was no association between the *VEGFA* polymorphisms and treatment response (*p* > 0.05).

In a pilot study by Dabir *et al*. [86], the authors conducted a gene expression analysis to identify biomarkers that distinguish bevacizumab responders from nonresponders. RNA from whole blood was assessed to identify systemic gene expression signatures relevant to treatment response. The Agilent Human Gene Expression microarray kit was used to generate gene expression data. Analysis of bevacizumab responders (*n* = 5) versus nonresponders (*n* = 5) identified 61 differentially expressed genes (2.5-fold change), 35 of which were upregulated and 26 downregulated. The majority of differentially expressed genes, both up and downregulated, were present in transcription regulation (*n* = 25) or receptor activation (*n* = 21) pathways. However, due to the very limited number of samples in these analyses and no comparison of the same individuals before receiving bevacizumab treatment, the results need to be interpreted with caution.

Recently, Toraiha et al. [87] conducted a study on a Middle-East population exploring the relationship between levels of serum hyperglycemia-related long noncoding ribonucleic acids (lncRNAs) and response to anti-VEGF injection (aflibercept). LncRNAs encode RNA transcripts longer than 200 nucleotides, and despite not being translated into protein, they are capable of regulating several critical biological processes. There is a growing body of evidence implicating lncRNAs in various pathological conditions, including DR. However, Toraih *et al*. [87] found no association with aflibercept response (DME = 75, responder = 51, nonresponder = 9, missingdata = 15) and circulating levels of hyperglycemia-related lncRNAs, including retinal noncoding RNA 2, nuclear-enriched abundant transcript 2, cyclin-dependent kinase inhibitor 2B antisense RNA 1, and plasmacytoma variant translocation 1. In contrast to other studies, this study evaluated the treatment response only after a single dose of intravitreal aflibercept, which may not be sufficient to induce robust gene expression changes in the circulation. Evidence shows that not all patients benefit after a single dose of anti-VEGF and it is advisable to wait until at least 3-4 monthly injections have occurred before defining treatment response [89, 90]. Furthermore, the study failed to define the response criteria clearly.

## 5. Conclusions

A major concern with most studies of DME genetics to date relates to the size of the study cohort. Some studies were conducted with a sample size of less than 50 [15, 86], and caution is warranted when interpreting the results. Even in larger DME cohorts, only nominal associations with genetic polymorphisms were detected [46, 74], not reaching robust statistical significance. It should be noted that there is significant overlap in phenotype between DME and other subtypes of DR. Many patients display multiple phenotypes and attempts to separate and analyze only DME might not always be feasible and practical, contributing to small sample sizes. The spectrum of overlapping DR and DME phenotypes makes it extremely challenging to distinguish genetic effects of relevance to specific subtypes, and also potentially creates heterogeneity when attempting to analyze phenotypes as a group.

Comparing results for specific genes between studies is complicated considering that for most genes, there was no single polymorphism or genetic model consistently investigated. With the exception of the -643G > C variant in *VEGFA*, none of the studies performed replication analyses in an independent cohort, nor directly replicated findings from other studies. In the handful of published treatment response studies, there was striking variation in the follow-up period, the definition of response, and a different anti-VEGF agent was used in each study. These factors also make meta-analysis of multiple studies extremely challenging. Consideration should be given to including commonly used definitions as well as treatment and follow-up regimes when designing new studies to evaluate the genetics of treatment response in DME.

In summary, the role of inherited genetic polymorphisms in DME development and treatment response is still poorly understood, with a paucity of dedicated, well-powered studies in this field. Given the social and economic burden of DME and its impact on an individual's visual morbidity, genetic studies of larger, homogeneous patient cohorts are warranted, including meta-analysis of multiple studies where appropriate. Such studies will not only lead to a greater understanding of DME but may also impact clinical practices including better screening of at-risk populations and distinguishing patients who are more or less likely to respond to anti-VEGF agents. Robust genetic findings may even identify new therapeutic targets to complement and extend the success seen with anti-VEGF agents.

## Figures and Tables

**Table 1:** Candidate genes evaluated for association with diabetic macular edema.

Gene	Chr	Cohort size	DM type	Country	Variant	*p* value	Reference
*APOE*	19	DME = 36 Healthycontrols = 22^∗^	T2	Mexico	*ε*2, *ε*3, *ε*4	*p* < 0.05	Santos et al. [15]
eNOS	7	DME = 48 DRwithoutDME = 69^∗^	T2	Japan	-786T>C (rs2070744) 27-bpVNTR Glu298Asp (rs1799983)	*p* = 0.029 *p* = 0.006 *p* > 0.99	Awata et al. [18]
eNOS	7	DME = 100 DRwithoutDME = 87^∗^	T2	South India	27-bpVNTR	*p* > 0.05	Uthra et al. [19]
MnSOD	6	DME = 37 DRwithoutDME = 93^∗^	T2	South Korea	Ala16Val (rs4880)	*p* < 0.05	Lee et al. [25]
EPO	7	CombinedDME = 90 (DMwithoutDR = 233^∗^) T1DME = 24 (DMwithoutDR = 67^∗^) T2DME = 66 (DMwithoutDR = 166^∗^)	T1+T2	Australia	rs1617640 rs507392 rs551238	For all three SNPs *p* = 0.018 (T2) *p* = 0.040 (T1 + T2)	Abhary et al. [30]
VEGFA	6	DME = 63 DRwithoutDME = 112^∗^	T2	Japan	-2578C>A (rs699947) -1154G>A (rs1570360) -634C>G (rs2010963)	*p* = 0.148 *p* > 0.999 *p* = 0.047	Awata et al. [37]
VEGFA	6	DME = 64 DRwithoutDME = 148^∗^	T2	Egypt	-634C>G (rs2010963)	*p* = 0.019 (genotype) *p* = 0.022 (allele)	Shazly et al. [40]
VEGFA	6	CombinedDME = 93 DMwithoutDR = 281^∗^	T1 + T2	Australia	rs699946 rs833068 rs10434	*p* = 0.039 (T1) *p* = 0.017 (T1) *p* = 0.027 (T2) and *p* = 0.003 (T1 + T2)	Abhary et al. [36]
VEGFC	4	CombinedDME = 425 (DRwithoutDME = 952^∗^) T1DME = 64 (DRwithoutDME = 241^∗^) T2DME = 361 (DRwithoutDME = 711^∗^)	T1 + T2	Australia	rs17697515 rs17697419 rs2333526	*p* = 0.004 (T2) and *p* = 0.009 (T1+T2) - -	Kaidonis et al. [46]
PEDF	17	DME = 66 DMwithoutDR = 229^∗^	T2	Japan	rs12150053 rs12948385	*p* = 0.004 *p* = 0.008	Iizuka et al. [50]
*ALR2*∗	7	Proliferative DR with DME = 20 Non-proliferative DR with DME = 35 Proliferative DRwithoutDME = 35 Non-proliferative DRwithoutDME = 15	T2	South India	(CA)n (Z-2) allele	*p* < 0.05	Kumaramanickavel et al. [59]
MiRNA-146a	5	Combined DME = 1026 T1DME = 170 (no/minimalDR = 258^∗^) T2DME = 856 (no/minimalDR = 895^∗^)	T1 + T2	Australia	rs2910164	*p* = 0.025 (T2)	Kaidonis et al. [64]
MiRNA	5	DME = 89 (DMwithoutDR = 228^∗^)	T1	Australia	rs10061133 rs1049835	- -	Liu et al. [65]
CA	8	DME = 93 DMwithoutDR = 235^∗^	T1+T2	Australia	10 tag SNPs across *CA* gene	*p* > 0.05	Abhary et al. [68]
MCP1∗	17	DME = 446 (mildDME = 207, moderateDME = 173, severeDME = 66)	T2	North China	-2518A>G (rs1024611)	*p* > 0.05	Dong et al. [69]
*CXC* chemokine family∗	4	DME = 446 (mildDME = 207, moderateDME = 173, severeDME = 66)	T2	North China	-251T>A in CXCL8 -1596C>T in CXCL10	*p* > 0.05 *p* > 0.05	Dong et al. [70]
*SLMAP*	3	DME = 49 DMwithoutDR = 160^∗^	T2	Qatar	rs17058639	*p*_trend_ = 0.0425	Upadhyay et al. [71]

Chr: chromosome; DM: diabetes mellitus; DR: diabetic retinopathy; DME: diabetic macular edema; T1: type 1 diabetes mellitus; T2: type 2 diabetes mellitus; APOE: apolipoprotein E; EPO: erythropoietin; eNOS: endothelial nitric oxide synthase; VEGFA: vascular endothelial growth factor A; VEGFC: vascular endothelial growth factor C; MnSOD: manganese super-oxide dismutase; MiRNA: micro-ribonucleic acid; ALR2: aldose reductase 2; PEDF: pigment epithelium growth factor; CA: carbonic anhydrase; MCP1: monocyte chemoattractant protein 1; CXCL8: C-X-C motif chemokine ligand 8 (also known as interleukin 8, IL8); CXCL10: C-X-C motif chemokine ligand 8 (also known as interferon-inducible cytokine IP10); SLMAP: sarcolemma associated protein; ^∗^controls; ∗subtypes of DME were compared with each other.

**Table 2:** Summary of studies evaluating the association of genetic variants and changes in gene expression with response to anti-VEGF therapies in diabetic macular edema.

Gene and variants	Anti-VEGF drug	DM type	Response criteria	Cohort size	Follow-up (months)	*p* value	Country	Reference
*VEGFA* -634C>G (rs2010963)	Bevacizumab	T2	Non-responder: increase in VA < 3 lines AND <50% decrease in CMT Responder: increase in VA ≥ 3 lines AND ≥50% decrease in CMT	64 DME Responder = 36eyes (24 patients) Non − responder = 68eyes (40 patients)	9-12	*p* < 0.001	Egypt	Shazly et al. [40]
*VEGFA* rs2010963 rs2146323 rs10434 rs833069 rs6921438	Ranibizumab	NA	Non-responder: increase in VA < 2 lines AND CMT > 300 microns Responder: increase in VA ≥ 2 lines AND CMT ≤ 300microns	95 DME Responder = 53 Non − responder = 42	5	*p* > 0.05 for all variants	Turkey	Tetikoğlu et al. [85]
Transcriptome-wide gene expression analysis1	Bevacizumab	T2	Non-responder: stable/worsening/<10% reduction in CMT Responder: >10% reduction in CMT	Responder = 5 Non − responder = 5	NA	35 genes upregulated 26 genes downregulated	India	Dabir et al. [86]
Expression of multiple lncRNA genes2	Aflibercept	T2	Not defined	75 DME Responder = 51 Non − responder = 9 (missingdata = 15)	1	*p* > 0.05 for all lncRNAs	Egypt	Toraih et al. [87]

Study evaluated mRNA expression in peripheral whole blood following bevacizumab treatment. Study evaluated serum levels of hyperglycaemia sensitive long non-coding RNA following aflibercept treatment. DM: diabetes mellitus; NA: not available; DME: diabetic macular edema; VA: visual acuity; CMT: central macular thickness; VEGFA: vascular endothelial growth factor A; lncRNA: long non-coding ribo-nucleic acid.
